# Risk factors for equine strangulating lipoma colic: An international, case–control study

**DOI:** 10.1111/evj.70104

**Published:** 2025-10-08

**Authors:** Alexandra Gillen, Diana Hassel, Sam Gonzalez, Victoria Savage, Anje Bauck, David Freeman, Debra C. Archer

**Affiliations:** ^1^ Philip Leverhulme Equine Hospital University of Liverpool Cheshire UK; ^2^ Colorado State University Veterinary Teaching Hospital Colorado State University Fort Collins Colorado USA; ^3^ Three Counties Equine Hospital Gloucestershire UK; ^4^ College of Veterinary Medicine University of Florida Gainesville Florida USA; ^5^ Present address: Idaho Equine Hospital Caldwell Idaho USA

**Keywords:** colic, equine metabolic syndrome, horse, laminitis, risk factors, strangulating lipoma obstruction

## Abstract

**Background:**

Obstruction by strangulating lipoma (SLO) is one of the most common causes of small intestinal strangulation in horses and is fatal without surgery. Current knowledge of risk factors for SLO is limited to horse signalment features. To date, other risk factors have not been investigated.

**Objectives:**

To investigate horse‐ and management‐level risk factors for SLO using a population of healthy horses as controls.

**Study Design:**

Matched, case–control study.

**Methods:**

A prospective, international multicentre study was conducted in the UK and USA between January 2022 and May 2024. Cases were horses with SLO confirmed at exploratory laparotomy at the four participating hospitals. Three controls per case were randomly selected, matched on clinic and time. Conditional logistic regression was used to identify associations between variables and the risk of SLO.

**Results:**

Data from 55 SLO cases (UK, *n* = 42; USA, *n* = 13) and 167 matched controls were analysed. In a final multivariable model, increased age (odds ratio [OR]: 1.15; 95% confidence intervals [95% CI]: 1.04–1.28; *p* = 0.008) and particular breeds (Pony, Welsh Section D/Cob, American Quarter Horse/American Paint Horse/Appaloosa/Arabian) were at increased risk of SLO (OR: 1.53; 95% CI: 1.10–2.15; *p* = 0.012). SLO was more likely in horses with a prior history of laminitis (OR: 10.94; 95% CI: 2.21–54.13; *p* = 0.003) or increased stabling in the previous 4 weeks (OR: 6.79; 95% CI: 1.96–23.54; *p* = 0.003). Management strategies to maintain optimal weight and address equine metabolic syndrome were protective (OR: 0.19, 95% CI: 0.39–0.94; *p* = 0.041).

**Main Limitations:**

Potential for selection and recall bias by horse owners.

**Conclusions:**

This study adds to knowledge of the epidemiology of SLO and has identified new risk factors which may be modifiable. Strategies to prevent endocrine‐associated laminitis, including weight management in high‐risk groups, should be considered to minimise SLO risk.

## 1 INTRODUCTION

Obstruction by strangulating lipoma (SLO) is one of the most common causes of surgical colic,^1^ and is typically fatal without surgery.^2^, ^3^, ^4^, ^5^ The current lack of knowledge regarding potentially modifiable risk factors for SLO limits advice that can be given to owners of horses and ponies that are at higher risk of SLO. Knowledge of risk factors for SLO could aid the understanding of disease causality and be used to devise disease prevention strategies. In addition, knowledge of risk factors is also important to assist early identification of cases by primary care veterinary surgeons and referral centres, resulting in exploratory laparotomy at an earlier stage, and therefore an improved prognosis.^6^

Currently, investigation of risk factors for SLO has been restricted to signalment features,^3^, ^4^ which are nonmodifiable. Horses of greater age have been shown to be at increased risk of SLO in several studies,^3^, ^4^, ^7^, ^8^ with SLO cases having a mean age of 16–19 years. Geldings have also been identified to be at around double the likelihood of SLO compared to females.^3^, ^7^, ^9^ Certain breeds including ponies,^4^ Saddlebreds, Arabians^7^ and American Quarter Horses^10^ are also at increased risk of SLO. However, these findings are largely based on studies that have used other colic or hospital cases as controls,^3^, ^4^, ^7^ introducing potential for population bias and exposure of controls to risk factors for other types of colic or reasons for hospitalisation.

Results of multiple epidemiological investigations have demonstrated that colic is multifactorial in nature and that specific risk factors exist for particular types of colic.^10^, ^11^ Factors that predispose to lipoma formation, such as increased adiposity, would appear to be plausible potential risk factors; however, this is an area in which limited investigation has been undertaken. Increased weight and body condition score (BCS) have been suggested to be risk factors for SLO; however, this was not confirmed in a retrospective study.^7^ In addition, a cadaver study found that both insulin dysregulation (measured by serum glucose and insulin) and histological evidence of pituitary hyperplasia were associated with the presence of mesenteric lipomata.^12^ Therefore, investigation of markers of adiposity and endocrine disease, including equine metabolic syndrome (EMS), on the risk of SLO is justified.

The aim of this study was to identify horse‐ and management‐level risk factors for SLO using a population of non‐hospitalised controls. The hypotheses were that (1) increasing age, male sex and pony breeds; (2) endocrine and metabolic disease and (3) horses exposed to recent management changes would be at increased risk of SLO.

## 2 MATERIALS AND METHODS

### 2.1 Study design

A prospective multi‐centre, international, matched, case–control study was conducted between 2022 and 2024 to identify associations between various horse‐ and management‐level risk variables and SLO (outcome variable). Four equine clinics, two in the United Kingdom (one university‐based, one private practice) and two in the United States (both university‐based), participated in the study.

Sample size estimation was performed using G*power (version 3.1.9.7; Bonn University). For EMS as the exposure of interest (exposure in the United States and United Kingdom 18%–23.3%^13^, ^14^, ^15^), a study with 41 cases and three controls per case, assuming 18% exposure (EMS) in controls,^13^, ^14^, ^15^ would have 80% power to detect odds ratios (ORs) of 2.5 or higher with 95% confidence intervals (95% CIs). A ratio of 3:1 controls to cases was used. Matching on clinic and time was used to control for differences in horse and management‐level factors between different geographic regions.^16^, ^17^, ^18^

### 2.2 Case and control definition and recruitment

Principal investigators (PIs) at each site were responsible for the recruitment of eligible cases and controls. Cases were defined as horses or ponies (‘horses’) presented to participating clinics for surgical management of acute colic and where SLO was confirmed at laparotomy. Once informed owner consent had been obtained by the relevant PI and clinic team, the questionnaire (Survey S1) was conducted as soon as possible after surgery (one to 7 days) depending on individual clinic and client wishes.

A list of clients seen at each of the collaborating clinics in 2019, 2021 and 2022 was generated; the year 2020 was excluded due to COVID‐19 limiting hospital admissions to emergency cases during specific times and according to national/regional regulations. The study control population therefore was all horses currently owned or cared for by clients who attended one of the participating hospitals during 2019, 2021 or 2022. Control owners were randomly selected and asked to select one horse from their ownership who met the criteria below. To be eligible as a control, horses had to be aged more than or equal to 8 years of age,^3^, ^4^ as SLO has not been previously identified in horses younger than 8 years of age. The control owner was also required to confirm that the horse would be referred to the hospital for surgical management of acute colic if required. Essentially, control horses were representative of the at‐risk population and could have been a ‘case’ had SLO developed.^16^ Exclusion criteria were a previous diagnosis of SLO and horses that had exhibited colic signs in the previous 4 weeks. A control meeting the above criteria was then randomly selected from a list of eligible horses for each client (Data S1), and the questionnaire (Survey S1) was administered in the same way as for case horses.

### 2.3 Questionnaire design

A questionnaire (Survey S1) was constructed using information from previous epidemiological studies investigating horse‐ and management‐level risk factors for colic.^10^, ^11^, ^19^, ^20^ Data were also collected for other variables considered to be biologically plausible as potential risk factors for SLO.^7^, ^12^ Horse‐level data collected included signalment (sex, age, breed, height and weight), prior/current medical conditions including colic, laminitis, EMS, Pars Pituitary Intermedia Dysfunction (PPID) and stereotypic behaviours. Management data collected included: stabling (stalling) and pasture turnout routines, feeding, exercise and recent changes in management (previous 4 weeks). Data were obtained around owner/carer observations of individual horses' ability to gain/lose weight and current weight management strategies. Veterinary interventions and medications administered in the previous 4 weeks were also recorded. All data were entered onto Joint Information Systems Committee (JISC) (version 2) prior to data being exported as a csv. file (Numbers, Apple Inc).

### 2.4 Data collection

Questionnaires were conducted over the telephone by the PI or a nominated member of the clinic team, or by the chief investigator if requested by Centres 2, 3 and 4 (AG). Owners/carers of both cases and controls also had the option to complete the questionnaire online via a survey link (JISC). All case questionnaires were conducted within 1 week of the SLO case occurring. All control questionnaires were conducted within 4 weeks of their matched SLO case.

### 2.5 Data analysis

Statistical analyses were performed in Stata (Intercooled Stata 18.0, StataCorp LLC). Variables were screened using a conditional univariable logistic regression model with SLO as the dependent variable. Missing categorical data were coded to allow complete case analysis.^21^ Continuous variables were assessed for normality using a Shapiro–Wilk test, and marginal analyses were utilised to assess their functional relationship with SLO and evidence of non‐linearity. Continuous variables were also evaluated in quintiles, quartiles and other biologically plausible categories. To reduce the effects of collinearity, continuous variables were centred by subtracting the mean of the variable from all recorded observations.^22^ Where necessary for analysis, categories within a variable were grouped providing this was biologically plausible. Correlation between variables was assessed using a Spearman or Pearson correlation coefficient, Kendall's tau, or rank biserial correlation coefficient.^23^ Where variables were strongly correlated (*r* > 0.7), variables were selected based on statistical significance or biological plausibility or were grouped together as appropriate. Variables with a univariable *p* < 0.2 were selected for inclusion in a multivariable model. The model was built using a backwards stepwise approach where variables were retained in the model if their manual exclusion resulted in a likelihood ratio test statistic of *p* < 0.05.^24^ A change in the co‐efficient of >25% was considered indicative of confounding. The effect of biologically plausible interactions was tested in the model.

## 3 RESULTS

### 3.1 Descriptive analysis

**FIGURE 1:**
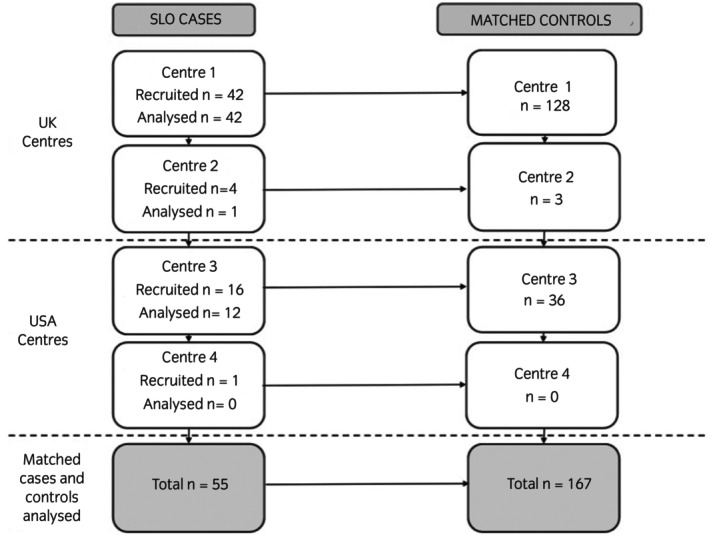
Flow chart showing recruitment of strangulating lipoma obstruction (SLO) cases and controls by centre. Cases were included in the matched analysis if matched controls meeting the inclusion criteria were available.

Over a 29‐month period (January 2022 to May 2024), 62 cases of SLO were identified and recruited onto the study. The mean age of SLO cases was 18.24 years (SD 4.00 years), the youngest being 9 years of age. Male horses accounted for 74.2% of SLO cases (*n* = 46 geldings, *n* = 1 stallion). Breed groups with SLO were Thoroughbred and Thoroughbred crosses (*n* = 3, 4.38% [Centre 1, *n* = 3]), Warmblood, Irish Draught and their crosses (*n* = 8, 12.9% [Centre 1, *n* = 8]), Ponies (*n* = 14, 22.6% [Centre 1, *n* = 13; Centre 2, *n* = 1]), Welsh Section D and Cob (*n* = 20, 32.3% [Centre 1, *n* = 15; Centre 2, *n* = 3; Centre 3, *n* = 2]), American Quarter Horse, American Paint Horse and Arabian (*n* = 13, 20.9% [Centre 1, *n* = 2; Centre 3, *n* = 10; Centre 4, *n* = 1]) and other (*n* = 4, 6.5% [Centre 1, *n* = 2; Centre 3, *n* = 2]). A percentage of 99.1 questionnaires was administered by telephone and 0.9% (*n* = 2, both controls) was completed online. Cases of SLO that did not have matched controls were excluded from the matched case control analysis. Three controls were obtained for each case with the exception of two SLO cases where four controls were recruited. Data from 55 SLO cases and 167 matched controls were therefore analysed using conditional logistic regression (Figure 1).

### 3.2 Univariable analysis

The results of univariable conditional logistic regression of all categorical and continuous variables with a *p* < 0.2 are shown in Table S1. Categorical and continuous variables with a *p* < 0.05 are shown in Tables S2 and S3, respectively.

**FIGURE 2:**
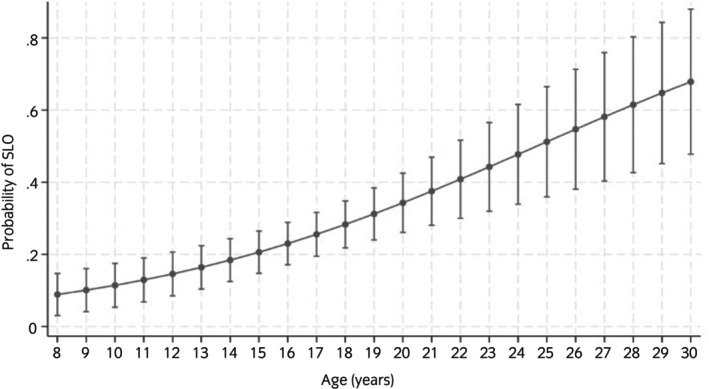
A profile plot demonstrated that a linear fit for age and the probability of strangulating lipoma obstruction (SLO) was appropriate. The plot shows the fitted curve with 95% confidence intervals.

Increasing age was significantly associated with SLO (*p* < 0.001) and this was linear in nature (Figure 2). Male horses were also significantly associated with increased likelihood of SLO (*p* = 0.017) as were certain breed groups (Ponies, Welsh Section D/Cobs, AQH/Paints/Appaloosas/Arabians; *p* = 0.01).

A diagnosis of laminitis or PPID was associated with an increased likelihood of SLO on univariable analysis (Table S2). A confirmed diagnosis of EMS was not significantly associated with an increased likelihood of SLO. Low data numbers in some categories, for example, horses with PPID that were not receiving pergolide (or equivalent medication) (*n* = 2), and horses with EMS that were receiving metformin (*n* = 1) precluded statistical analyses of any potential effect of pergolide or metformin administration in horses with PPID or EMS.

A number of risk factors previously identified to alter the risk of colic (in general and for specific forms of colic) were not significantly associated with the likelihood of SLO. There was no significant association between a prior history of colic,^25^ abdominal surgery^26^ or dental abnormalities,^10^ duration of time at pasture or stabled (stalled),^27^ quantity and types of feed,^25^ stereotypic behaviours^19^ and SLO risk.

### 3.3 Multivariable analysis

A multivariable model is shown in Table 1. Increasing age was strongly associated with a likelihood of SLO, the odds increasing by 15% for each additional year of age (*p* = 0.008). Ponies, Welsh Section D/Cob and American Quarter Horse/American Paint Horse/Arabian breed groups were at increased risk of SLO compared to Thoroughbred and Thoroughbred crosses (OR: 1.53; 95% CI: 1.10–2.15; *p* = 0.012). Sex of the horse was not retained in the final model when management‐level risk factors were included. A change in the duration (either an increase or decrease) of stabling in the previous 4 weeks was associated with an increase in the likelihood of SLO (*p* = 0.003). Horses with a prior history of laminitis were also more likely to have SLO (*p* = 0.003) compared to horses than had never been diagnosed with laminitis, even when adjusted for age. Horses that were currently undergoing management strategies to control their weight, either due to obesity or suspected/confirmed EMS had a reduction in likelihood of SLO (*p* = 0.04). No significant multiplicative interaction was found between variables in the final model. Sensitivity testing included the inclusion of centre or country (UK/USA) as a variable in the model. This did not result in a significant change in the variables retained in the final model.

**TABLE 1:** Multivariable logistic regression on 55 cases (SLO) and 167 matched controls evaluating horse–and management‐level risk factors for SLO.

Variable	Odds ratio	Standard error	95% confidence interval	*p* value
Age	1.15	0.06	1.04–1.28	0.008
Breed				
TB/TBx	Reference			
WBL/WBLx/ID/IDx	2.61	2.30	0.47–14.65	0.28
Pony	7.15	6.14	1.33–38.49	0.022
Welsh Section D/Cob	8.01	7.43	1.30–49.33	0.025
AQH/American Paint/Appaloosa/Arabian	10.60	11.49	1.29–87.06	0.028
Other	4.32	5.88	0.30–62.34	0.28
Not recorded				
Laminitis				
No	Reference			
Yes	10.94	8.92	2.21–54.13	0.003
No recorded	6.49	0.15	0.04–0.94	0.12
Management change for EMS/weight				
No	Reference			
Yes	0.19	0.15	0.04–0.94	0.04
Stabling change in previous 4 weeks				
No	Reference			
Yes	6.79	4.31	1.96–23.54	0.003
Not recorded				

Abbreviations: EMS, equine metabolic syndrome; PPID, pars pituitary intermedia; TB/TBx, Thoroughbred/Thoroughbred cross; WBL/ID/WBLx/IDx, Warmblood/Irish Draught/Warmblood Cross/Irish Draught cross.

## 4 DISCUSSION

This is the first study to investigate horse‐ and management‐level risk factors for SLO, using a representative population of at‐risk horses as controls. We have shown that specific risk factors exist for SLO colic, strengthening evidence of its association with ageing and breed type and furthering our knowledge around colic causality. Increased likelihood of SLO in horses with a history of previous laminitis as well as a protective effect of EMS/weight management strategies are novel findings. This information, together with the increased risk associated with recent changes in stabling, can be used to devise evidence‐based strategies to reduce the likelihood of SLO in high‐risk horses. This knowledge, in addition to the information obtained during the standard colic evaluation, can also be used by veterinary professionals and horse owners to assist early recognition and surgical management of horses with SLO to optimise patient outcomes.

The increasing prevalence of obesity in managed horse populations and its association with EMS and endocrinopathic laminitis has important implications for equine health and welfare.^28^, ^29^, ^30^, ^31^ This information should be taken into account when advising owners on preventive strategies. The finding in the present study that a prior history of laminitis was associated with increased risk of SLO has not been previously identified. In addition, this study has shown that management strategies to maintain an optimal weight, and strategies that were implemented as part of veterinary management of diagnosed or suspected EMS, were associated with reduced risk of SLO. The importance of management to control EMS has been demonstrated in the equine and human literature.^30^, ^31^, ^32^ Maintaining optimal equine weight throughout life to reduce EMS and endocrinopathic laminitis risk, particularly in older horses and ponies of certain breeds and those that have had a laminitic event previously, would also appear to be important to reduce the risk of colic due to SLO.

Increasing age has previously been identified as a risk factor for SLO utilising horses undergoing exploratory laparotomy for colic,^4^ horses hospitalised for colic,^3^ or for any reason^7^ as controls. The findings of the present study are consistent with the aforementioned studies and have strengthened the evidence of this important association with SLO, using a non‐hospitalised equine population as controls. Pedunculated lipomata, which are more likely to cause SLO compared to non‐pedunculated lipomata,^33^ are likely to take time to form,^34^ and may explain why SLO is rarely seen in horses aged less than 8 years of age. However, by adjusting for age in a multivariable analysis, we are able to demonstrate that whilst age is an important risk factor for SLO colic, other non‐age‐related factors are also associated with increased disease risk.^35^

Consistent with previous studies, we have also confirmed a breed association with SLO colic risk. Increased prevalence or risk of SLO has been reported in pony breeds,^4^ Quarter Horses^36^ and Arabians^7^ and reduced risk reported in Thoroughbreds.^4^ However, the aforementioned studies utilised horses undergoing exploratory laparotomy^4^ or hospitalised horses in general^7^, ^36^ as controls, which may introduce bias. The reason for this breed associated risk of SLO may be due to breed predisposition to increased BCS,^35^ and potential mesenteric/omental lipoma formation.^7^ Weight and markers of adiposity including BCS have been evaluated in a previous study and although specific markers of adiposity did increase the odds of lipomata development, they did not affect the odds of SLO.^33^ Alternatively, there may be breed‐related factors affecting adipose deposition and insulin regulation.^37^ These factors all merit further research.

Inconsistent with previous studies, including an unmatched case‐control study conducted by the authors using hospital‐based controls,^3^, ^4^, ^7^, ^33^ there was no association between geldings/stallions and SLO risk. Sex of the horse, whilst significantly associated with increased risk of SLO in the univariable analysis, dropped out of the model when laminitis and/or a change in stabling were included. Laminitis does not appear to have a sex predisposition,^38^ and no significant interaction between sex and laminitis was evident in this study. In people, although mesenteric lipomata are rare, both mesenteric lipomata and certain types of lipomata associated with the gastrointestinal tract occur more commonly in male compared to female patients.^39^, ^40^ Further investigation into potential genetic and hormonal factors involved in lipomata and SLO development also appears to be justified.

This is the first time that a history of prior laminitis, a potentially modifiable risk factor, has been shown to be a risk factor for SLO. This increase in risk was also identified in the univariable analysis in horses that had laminitis in the previous 12 months, and in the previous 4 weeks. Laminitis was not correlated with age, a diagnosis of PPID, a diagnosis of EMS, or a change in stabling or management in the previous 4 weeks in the present study. Although a link between insulin dysregulation and lipomata has been proposed,^12^ a history of laminitis has not previously been assessed in SLO colic risk. One study has, however, found hoof growth rings scores, which have been validated as being indicative of laminitis within the previous 5 years,^13^ to be a risk factor for development of lipomata, using horses undergoing exploratory laparotomy as a control population.^33^ In the present study, although PPID was a risk factor in the univariable analysis for SLO, neither PPID nor EMS remained in the multivariable model. This suggests that uncontrolled or undiagnosed endocrine disorders (e.g., PPID or EMS), or carbohydrate‐induced laminitis, may place horses at higher risk of SLO, compared to PPID or EMS that has been diagnosed and is appropriately controlled.^28^, ^31^

Recent changes in management have been consistently identified as a risk factor for colic in general, and for specific types of colic.^10^, ^11^ This study has also identified a change in stabling duration in the previous 4 weeks as a risk factor for SLO. It is plausible that any management changes such as a sudden increase in stabling can result in altered motility of the small intestine and subsequent formation of small intestinal obstruction by a pedunculated lipoma.^41^ Epidemiological studies demonstrate that in general, colic risk is reduced by avoiding sudden changes in turnout and stabling, avoiding long periods of time spent stabled, and by providing consistent pasture turnout.^26^, ^42^, ^43^, ^44^, ^45^, ^46^ This study adds to the evidence that sudden increases in stabling should be avoided in horses at high risk of SLO.

A matched study design and analysis was used to control for known confounders, specifically, variation in breed types and management practices due to geographic and climate differences.^16^, ^17^, ^18^, ^47^, ^48^ This study was based on four hospital populations in the United Kingdom and United States to assist the generalisability of findings to other horse populations and to increase the speed of recruitment of a suitable number of cases and controls. However, due to time and resource limitations, the majority of cases and matched controls were from one UK hospital population. It must also be acknowledged that a larger study would be required to reduce the risk of confounding, despite the evaluation of a large number of risk factors. As with any questionnaire‐based studies, response and recall bias are potential issues.^49^, ^50^ Recall bias was minimised as best as possible by limiting the duration of time between SLO diagnosis or control selection and questionnaire administration to a maximum of 7 days. Some questions were not answered, either due to the owner not knowing the information or due to potential questionnaire fatigue.^49^ Due to the nature of the study, it was not possible to obtain specific validated measures of BCS/adiposity and EMS status in cases and controls.

This is the first study to investigate management‐level risk factors for SLO, and to use a healthy, at‐risk population of non‐hospitalised horses as controls. The study confirmed horses of increasing age and specific breed groups to be at the highest risk of SLO. Laminitis prevention, weight/EMS management and avoidance of sudden changes in stabling duration may be important ways to reduce the likelihood of SLO in high‐risk horses. Use of these new potential risk factors in the diagnosis of SLO should be applied carefully with other known risk factors. Further studies are warranted to investigate the association between lipoma formation and age, breed and endocrine status.

## FUNDING INFORMATION

This study was funded by the Arden and Claudia Sims Lipoma Foundation.

## CONFLICT OF INTEREST STATEMENT

The authors declare no conflicts of interest.

## AUTHOR CONTRIBUTIONS

**Alexandra Gillen:** Conceptualization; investigation; writing – original draft; funding acquisition; methodology; writing – review and editing; formal analysis. **Diana Hassel:** Investigation; methodology; writing – review and editing. **Sam Gonzalez:** Investigation; writing – review and editing. **Vicky Savage:** Investigation; writing – review and editing. **Anje Bauck:** Investigation; writing – review and editing. **David Freeman:** Conceptualization; investigation; funding acquisition; methodology; writing – review and editing. **Debra C. Archer:** Conceptualization; investigation; funding acquisition; writing – original draft; methodology; writing – review and editing; formal analysis; supervision.

## DATA INTEGRITY STATEMENT

Alex Gillen and Debra Archer had full access to all the data in the study and take responsibility for the integrity of the data and the accuracy of data analysis.

## ETHICAL ANIMAL RESEARCH

The study was approved by the University of Liverpool Veterinary Ethics Committee (VREC 1154). The study was also approved by the relevant ethics committees as CSU and UF.

## INFORMED CONSENT

Consent was obtained from all owners, via completion of consent form.

## Supporting information


**Data S1:** Matched study case control questionnaire.

**Data S2:** Control recruitment.

**Table S1:** Univariable analyses on 55 cases (SLO) and 167 matched controls evaluating horse‐ and management‐level risk factors for SLO.

**Table S2:** Univariable analyses of categorical variables on 55 cases (SLO) and 167 matched controls evaluating horse‐ and management‐level risk factors for strangulating lipoma obstruction (SLO).

**Table S3:** Univariable analyses of continuous variables on 55 cases (SLO) and 167 matched controls evaluating horse‐ and management‐level risk factors for strangulating lipoma obstruction (SLO).

## Data Availability

The data that support the findings of this study are available upon reasonable request from the corresponding author. Open data sharing exemption granted by the editor.
