# Supplementary material for: Risk factors for equine strangulating lipoma colic: An international, case–control study
Source: Equine Vet J. 2025 Oct 8;58(4):1016–23. doi: 10.1111/evj.70104 (PMC13244184; doi:10.1111/evj.70104)
Supplement: Supplementary file 1 — Data S1: Matched study case control questionnaire. [file EVJ-58-1016-s004.pdf]

Survey S1:

Matched Study Case Control Questionnaire

1. **Horse ID (unique study ID)**

2. **DATE OF QUESTIONNAIRE COMPLETION:**

Thank you in advance for helping with this study. Please note the following points:

- **All of the questions relate to the horse/pony mentioned above**
- **The word 'horse' defines both horses and ponies.**
- **This questionnaire is being used in both Europe and the USA, some spelling and terminology may appear less familiar.**
- **If you have any queries, please contact XXXX (**email)

SECTION A: HORSE DETAILS

3. **What age is this horse/pony?** years

4. **What is their breed?**

5. **What sex are they?**

Mare (not in foal)	
Mare (in foal)	
Gelding	
Stallion	

6. **What is the height of this horse?** hh or 0 0 0 cm **(please choose correct units)**

7. **What is their weight?** 0 0 0 0kgs or 0 0 0 0lbs **(please choose the correct units) and how was this determined:**

Actual Weight	
Weightape	
Guess	

8. **Who is the main carer for this horse? On livery**

Owner	
Yard manager and staff	
Loaner	
Carer	
Other (please state)	

9. **How many people care for this horse on an average day?** 00

SECTION B: MEDICAL AND HEALTHCARE HISTORY

10. **How long have you owned this horse? 00(years) or 00 (months)**

11. **Has this horse ever had laminitis? Check the correct box**

Yes	
No	
Don't know	

If no or don't know, go to question 12

a. **If yes, how many episodes (requiring pain relief eg. bute, flunixin, Danilon, Paracetamol) has this horse had within the last 12 months? 00**

If 0, go to question 13.

b. **Have there been any episodes (requiring pain relief) within the last 4 weeks?**

Yes	
No	
Don't know	

c. **Which of the following statements best describes the most severe laminitic signs shown during the last 12 months (please select only 1 box)?**

When standing, the horse shifted weight from one foot to the other. There was no lameness evident at a walk, but at the trot, there was a stilted gait/shortened stride	
The horse moved willingly at a walk but with a stilted gait. A foot could be lifted off the ground without difficulty	
The horse was reluctant to move and resisted attempts to lift one or all of the feet	
The horse would not move without being forced and would possibly lie down more than usual. It was almost impossible to lift a foot off the ground.	

12. **Has this horse ever been diagnosed with Equine Metabolic Syndrome (EMS)? EMS is an inability to regulate blood glucose and insulin leading to a predisposition to laminitis. Often, but not always, horses are overweight, and require minimal feed to maintain body condition.**

If no please go to Question 13

Yes (diagnosed by a veterinary surgeon/veterinarian)	
Yes (diagnosed without veterinary assistance as the horse has obvious signs of EMS)	
No	
Don't know / I have never heard of EMS	

a. **Roughly how long ago was the diagnosis made? 00 months or 00 years (choose the correct timescale)**

b. **Has this horse every been prescribed metformin?**

Yes (currently receiving metformin)	
Yes (but not currently receiving metformin)	
No	
Don't know	

13. **Is this horse currently undergoing dietary management for laminitis/EMS? Choose all that apply**

Yes: restricted hay/haylage	
Yes: restricted concentrates/hard feed	
Yes: restricted turnout	
Yes: low calorie feeds	
Yes: soaking hay	
Don't know	
No	
Yes (other) please state	

14. **Has this horse ever been diagnosed with Cushing's syndrome or Pituitary Pars Intermedia Dysfunction (PPID)?**

If no please go to Question15

Yes	
No	
Don't know	

a. **Roughly how long ago was the diagnosis made?** 00 **months or** 00 **years (choose the correct timescale)**

b. **Has the horse ever been prescribed pergolide/Prascend/Permax?**

Yes (currently receiving pergolide/Prascend/Permax)	
Yes (not currently receiving pergolide/Prascend/Permax)	
No	
Don't know	

15. **Has this horse had a dental examination in the last 12 months?** yes

Yes (performed by a veterinarian/veterinary surgeon)	
Yes (performed by a veterinary technician/qualified dental technician e.g. BAEDT trained)	
Yes (performed by an unqualified dental technician)	
Yes (unsure who performed the examination)	
No	
Don't know	

a. **Approximately how long ago was the last dental examination performed (if known):** 00 **weeks /** 7**months ago**

b. **Does this horse have any known dental issues?**

Yes	
No	
Don't know	

If yes please describe:

c. **Has this horse been observed to having difficulty chewing feed (quidding/dropping feed) in the last 4 weeks?**

Yes	
No	
Don't know	

16. **Has this horse received any form of vaccination in the last 4 weeks?** *Vac for tet*

Yes	
No	
Don't know	

If yes:

What was your horse vaccinated against	Approximate date given
	d d / m m m /yy
	d d / m m m/yy

17. **How often is this horse dewormed (wormed)?**

Never	
Depending on results of fecal worm egg count	
Every 6-13 weeks	
Every 14 weeks-6 months	
Less frequently	
Other or combination of above (please state)	

b.What product was the last dewormer / wormer the horse received and when?

Wormer brand / type	Date given
Equivet	d d / nov/ 2021 (2 months)
	d d / m m m / y y y y

c. **If tested, do you know what this horse's last known parasite results were (if not performed or unknown, go to question 18)? Not done**

Test	Date	Normal	High
Faecal worm egg count	m m m / y y y y		
Tapeworm ELISA	m m m / y y y y		

18. **Does this horse have any known ongoing medical problems (e.g. chronic lameness, skin conditions)?**

Yes	
No	
Don't know	

If Yes, please give brief details:

Category of condition	Check if applicable	Diagnosis/further details
Orthopaedic (orthopaedic)/lameness		
Respiratory		
Dental/gastrointestinal		
Weight loss		
Lethargy/fever		
Other (please state)		

19. **Has this horse required veterinary examination or treatment (excluding routine vaccination) in the last 4 weeks?**

Yes	
No	
Don't know	

If yes please give reason:

	Approximate date	Reason	Has this Resolved or is it ongoing	
			Resolved	Ongoing
**Reason 1**	d d / m m m /yy			
**Reason 2**	d d / m m m /yy			
**Reason 3**	d d / m m m /yy			
**Reason 4**	d d / m m m /yy			

20. Has this horse received any medications excluding wormers or vaccinations (e.g. antibiotics / anti-inflammatories) in the last 4 weeks?

Yes	
No	
Don't know	

If yes, please give details:

**Medication type**	**Product name (state unsure if necessary)**	**How was this given?** (in feed / intramuscular / intravenous / other)	**Number of days received** (put 1 if single administration of medication)	**Is your horse still receiving this?**	
				**Yes**	**No**
Sedation			00		
Pain relief			00		
Antibiotic			00		
Steroid			00		
Other (please state)			00		
Other (please state)			00		
Other (please state)			00		

21. **Has this horse ever been known to have a colic episode?**

Yes	
No	
Don't know	

If yes:

Number of episodes (vet and non-vet attended) in the last 12 months:	0 0
Number of veterinary visits for colic in the last 12 months:	0 0
Date of the last colic episode (if known)	d d / m m m / y y y y

22. **Has this horse ever undergone abdominal surgery** (e.g. colic, ovariectomy, caesarean section)**?**

Yes	
No	
Don't know	

If yes:

**Date of surgery** (if known / or approximate date)	**Problem identified at surgery** (if known)
d d / m m m / y y y y	
d d / m m m / y y y y	

SECTION C: MANAGEMENT DETAILS

23. What is this horse's primary use when in good health (please select one option)?

Pasture companion	
Young child's riding pony	
Hacking	
Schooling	
Riding school	
Showing	

Dressage	
Showjumping	
Eventing	
Hunting	
Driving	
Western pleasure	
Barrel racing	
Reining, cutting, roping, other rodeo event	
Racing	
Other (please state)	

24. How many days per week is the horse currently being exercised? 0

25. For how many hours per week is the horse exercised 0

26. **What is the horse's current level of activity?**

Retired or is not exercised	
Low intensity: eg, hacking, light schooling	
Moderate intensity: eg. schooling, dressage, occasional jumping, driving	
High intensity: eg, racing, eventing at competition level, show jumping at competition level, rodeo,	

If the selected horse is retired or is not exercised, please go to Question 27

a. **Have there be any changes in exercise duration or intensity within the last 4 weeks?**

Yes, exercise has increased	
Yes, exercise has decreased	
Yes, exercise has ceased with the horse on pasture turnout<br>Yes – other (please describe)	
Yes, exercise has ceased with the horse on box rest	
No change	

27. This question relates to weight management and is not related to diet management due to colic or other medical issues. Please choose the statement that best suits this horse.

Gains weight very easily and their diet has to be closely managed year-round to avoid them gaining weight	
Gains weight easily and close management of feed rations is required periodically	
No obvious change in weight throughout the year and no special diet management is required	
Struggles to maintain weight during the colder months and requires extra feed rations during this time	
Struggles maintain weight year-round and is often in reduced condition	

28. **How would you describe this horse's body condition according to what is optimum for health?**

Very overweight	
**Slightly overweight**	
**An appropriate weight**	
**Slightly underweight**	
**Very underweight**	
**Other (please state)**	

29. **How long is the horse currently stabled/stalled for per day?** 0 0 hours.

Please go to Question 29 is the answer is zero hours ??24 hrs

a. What bedding is the horse stabled on?

Straw	
Shavings	
Paper/cardboard	
Peat	

Rubber matting and no bedding	
Other: Please State	

b. Has this horse ever been observed to eat the bedding it is currently on?

Yes	
No	
Don't know	

30. How many hours per day, on average is this horse being turned out for currently?

0 0 . 0 hours / day maybe zero

a. What type of turnout is this?

Not applicable – not turned out	
Lush grass paddock	
Good condition grass paddock	
Bare grass paddock	
Yard arena	
Sand arena	
Other: Please state	

b. Approximately how many hours are they turned out at pasture per day in summer/winter? (if never on pasture, please write 0 hours)?

Summer e.g. mid July: 24 0 hours / day

Winter e.g. mid December0 . 0 hours / day

c. Do you do anything to modify feed availability or exercise when turned out?

**No**	
**Yes: tracking system**	
**Yes: strip grazing**	

**Yes: muzzle**	
**Yes (other) Please state**	
**Don't know**	

d. Is this horse routinely turned out with other horses

**Yes**	
**No**	
**Don't know**	

If no or don't know, go to question 31

How many other horses is this horse turned out with? 00

i. How would you rank this horse in terms of dominance within the group?

**Not applicable**	
**Dominant (the horse gets to any feed first and will chance others away from the feed)**	
**Mid-hierarchy (the horse has access to feed when desired as he/she is not chased off the feed but he/she does not typically reach the feed first)**	
**Bottom of the heirarchy (the horse is bottom of the herd rank and is chased away from feed by others)**	
**Don't know**	

31. What type of forage is this horse currently receiving? (please choose all that apply)

Dry hay (please state hay type)	
Soaked hay (please state hay type and duration (hrs) for which it is soaked)	
Haylage	
Grass only	
Other: Please state	

32. What type of concentrate / hard feed is this horse currently receiving? None

Brand/Feed type	Approximate weight/number of scoops in total per day	Check appropriate scoop size if weight not known			
		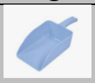	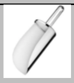	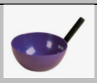	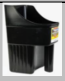

33. Do you feed any supplements, including fruit and vegetables?

Yes	
No	
Don't know	

If no, go to question 34

a. Please state the supplements given (including fruit and vegetables) and the quantity per day

Supplement/fruit/vegetables/SmartPak/Other	Quantity per day (If the supplement comes with a scoop, please state the number of scoops; if you feed a SmartPak, please state the supplements included)
Omega3	
Another suppl	

34-36. Has this horse had any changes in management relating to change in stabling/turnout, feeding or other factors such as transport in the last 4 weeks? Please complete the table below

**STABLING / TURNOUT**	**Change?** **Yes / No / Don't know**	**↑ time**	**↓Time**	**Other change e.g type (describe)**	**Days since change**	**Reason**
Bedding type					0 0	
Stabling					0 0	
Pasture Turnout					0 0	
					0 0	
**FEEDING**	**Change?** **Yes / No**	**↑ volume**	**↓Volume**	**Change in batch or type** **(No – Yes, describe)**		**Reason**
Forage	y			Accidental haylage 2 weeks ago	0 0	accidental
					0 0	
Concentrates					0 0	
					0 0	
Supplements in feed					0 0	
					0 0	
**OTHER MANAGEMENT / ROUTINE CHANGE (describe) e.g. transport, moved to different permises, staff changes due to sickness, etc**					**Days since change**	**Reason**
					0 0	
					0 0	
					0	

37. Does this horse display any stereotypic behaviours/behaviors ('stable vices')? Please complete the table below

Stereotypy	Click all the options that apply	Severity		
		Mild - seen occasionally – not seen daily	Moderate - seen daily but not for prolonged periods of time	Severe - seen daily and for prolonged periods of time
No stereotypy				
Crib biting				
Wind sucking				
Box walking				
Weaving	X			
Wood chewing				
Other: Please state				

Thank you for completing this questionnaire. If you have any queries, please feel free to contact XXXX at any time.
