# Supplementary material for: Risk factors for equine strangulating lipoma colic: An international, case–control study
Source: Equine Vet J. 2025 Oct 8;58(4):1016–23. doi: 10.1111/evj.70104 (PMC13244184; doi:10.1111/evj.70104)
Supplement: Supplementary file 2 — Data S2: Control recruitment. [file EVJ-58-1016-s005.pdf]

Data S1: Control Recruitment

- A list of dates during 2019, 2021 and 2022 were randomly generated using a random date generator (Random.org).
- Once notified of a SLO case recruited onto the study, the next date on the list was confirmed.
- The owner of the horse whose name was first in the alphabet on the designated day was contacted by email.
- In order to comply with GDPR, clients were initially contacted by the hospital they attended.
- Clients were then asked to select a horse from their current ownership who complied with the inclusion and exclusion criteria.
- If clients had more than 1 horse fitting the above criteria, they were asked to choose the horse with the name that was first alphabetically.
- If the client did not have any horses that fulfilled the criteria, they were excluded from the study.
- Owners were unaware of the hypotheses being tested.
