# Supplementary material for: Risk factors for equine strangulating lipoma colic: An international, case–control study
Source: Equine Vet J. 2025 Oct 8;58(4):1016–23. doi: 10.1111/evj.70104 (PMC13244184; doi:10.1111/evj.70104)
Supplement: Supplementary file 4 — Table S2: Univariable analyses of categorical variables on 55 cases (SLO) and 167 matched controls evaluating horse‐ and management‐level risk factors for strangulating lipoma obstruction (SLO). [file EVJ-58-1016-s001.pdf]

Table S2: Univariable analyses of categorical variables on 55 cases (SLO) and matched 167 controls evaluating horse- and management-level risk factors for strangulating lipoma obstruction (SLO).

Variable	Cases % (n)	Controls % (n)	Odds ratio	Standard error	95% Confidence interval	Significance
**Breed**						
**TB/TBx**	5.45 (3)	18.45 (31)			Reference	
**WBL/WBLx/ID/IDx**	14.55 (8)	33.33 (56)	1.53	1.11	0.37-6.31	0.56
**Pony**	23.64 (13)	15.48 (26)	5.49	3.77	1.43-21.07	0.013
**Welsh Section D/Cob**	32.73 (18)	13.10 (22)	9.17	6.59	2.24-37.49	0.002
**AQH/American/Paint/Appaloosa/Arabian**	16.36 (9)	14.88 (25)	4.80	3.90	0.97-23.63	0.54
**Other**	7.27 (4)	4.17 (7)	6.26	5.80	1.12-38.51	0.048
**Not recorded**	0 (0)	0.60 (1)				
**Sex**						
**Female**	23.64 (13)	42.26 (71)			Reference	
**Male**	76.36 (42)	57.74 (97)	2.38	0.87	1.17-4.86	0.017
**Laminitis (at any time)**						
**No**	50.91 (28)	90.48 (152)			Reference	
**Yes**	45.45 (25)	8,33 (14)	10.82	5.01	4.37-26.83	<0.001
**Unknown**	3.64 (2)	1.19 (2)	4.21	4.42	0.54-32.91	0.17
**Laminitis (previous 12 months)**						
**No**	56.36 (31)	92.26 (155)			Reference	
**Yes**	40.00 (22)	7.74 (13)	10.99	5.51	4.11-29.37	<0.001
**Not recorded/Unknown**	3.64 (2)	0 (0)				
**Laminitis (previous 4 week)**						
**No**	85.45 (14)	96.43 (162)			Reference	
**Yes**	12.73 (7)	2.98 (5)	4.30	2.52	1.36-13.57	0.013
**Not recorded/Unknown**	1.81 (1)	0.60 (1)	3	4.24	0.19-47.96	0.44
**Management changes for EMS**						
**No**	78.18 (43)	91.07 (153)			Reference	
**Yes**	21.82 (12)	8.93 (15)	2.95	1.29	1.25-6.94	0.014
**PPID**						
**No**	81.82 (45)	94.05 (158)			Reference	

**Yes**	18.18 (10)	5.95 (10)	4.10	2.16	1.46-11.51	0.007
**Worming frequency**						
**Dependent on FWEC**	20.00 (110	34.52 (58)			Reference	
**Less than every 6 weeks**	14.55 (8)	10.71 (18)	2.49	1.40	0.82-7.50	0.11
**Every 6 weeks to every 6 months**	29.09 (16)	40.48 (68)	1.27	0.58	0.52-3.09	0.60
**Less than every 6 months**	34.55 (19)	12.50 (21)	4.49	2,06	1.83-11.03	0.001
**Not wormed**	1.82 (1)	1.79 (3)	2.29	2.81	0.21-25.29	0.50
**Vet visit in the last 4 weeks**						
**No**	81.82 (45)	91.67 (154)			Reference	
**Yes**	18.18 (10)	8.33 (14)	2.62	1.24	1.04-6.62	0.042
**Number of vet visits**						
**0**	83.64 (46)	91.67 (154)			Reference	
**1**	12.73 (7)	3.57 (6)	3.55	1.99	1.19-10.64	0.02
**2**	3.64 (2)	4.76 (8)	0.90	0.75	0.18-4.58	0.90
**Owner's perception of weight loss/gain**						
**No change/loses weight easily**	40.00 (22)	55.36 (93)			Reference	
**Gains weight easily**	60.00 (33)	44.64 (75)	2.11	0.73	1.07-4.14	0.031
**Feed**						
**Chaff only**	18.18 (10)	26.19 (44)			Reference	
**Fibre feed (+/- chaff)**	34.55 (19)	21.43 (36)	2.31	1.04	0.95-5.57	0.06
**Mix (+/- chaff)**	36.36 (20)	39.39 (66)	1.32	0.57	0.56-3.07	0.52
**No hard feed**	1.82 (1)	2.38 (4)	1.10	1.28	0.11-10.81	0.94
**Not recorded**	9.09 (5)	10.71 (18)	1.22	0.77	0.264.20	0.75
**Change in last 4 weeks**						
**No**	65.45 (36)	79.76 (134)			Reference	
**Yes**	34.55 (19)	19.05 (32)	87.85	89.37	11.96-645.24	<0.001
**Not recorded**	0 (0)	1.19 (2)				
**Stabling change**						
**No**	70.91 (39)	94.64 (159)			Reference	
**Yes**	20.00 (11)	5.36 (9)	4.19	1.95	1.68-10.42	0.002
**Not recorded**	9.09 (5)	0 (0)				

**Use**						
**Retired**	27.27 (15)	17.86 (30)			Reference	
**Hacking**	45.45 (25	34.52 (58)	0.84	0.34	0.38-1.86	0.67
**Schooling/Jumping/Rodeo**	27.27 (15	47.62 (80)	0.39	0.16	0.17-0.87	0.022

TB/TBx = Thoroughbred/Thoroughbred Cross
WBL/ID/WBLx/IDx = Warmblood/Irish Draught/Warmblood Cross/Irish Draught Cross
EMS = Equine Metabolic Syndrome
PPID = Pars pituitary intermedia
FWEC = Faecal worm egg count
