# Supplementary material for: Risk factors for equine strangulating lipoma colic: An international, case–control study
Source: Equine Vet J. 2025 Oct 8;58(4):1016–23. doi: 10.1111/evj.70104 (PMC13244184; doi:10.1111/evj.70104)
Supplement: Supplementary file 5 — Table S3: Univariable analyses of continuous variables on 55 cases (SLO) and 167 matched controls evaluating horse‐ and management‐level risk factors for strangulating lipoma obstruction (SLO). [file EVJ-58-1016-s003.pdf]

Equine Veterinary Journal BEVA

Table S3: Univariable analyses of continuous variables on 55 cases (SLO) and 167 matched controls evaluating horse- and management-level risk factors for strangulating lipoma obstruction (SLO).

Variable	Missing cases % (n)	Missing controls % (n)	Odds ratio	Standard error	95% Confidence Interval	Significance
**Age**	0	0	1.17	0.46	1.08-1.26	<0.001
**Height**	0	0	0.95	0.01	0.93-0.98	0.001
**Length of Ownership**	0	0	1.08	0.03	1.02-1.15	0.006
**Time since PPID diagnosis**	0	0	0.99	0.0005	0.99-0.99	0.005
